# Antimicrobial Effects against Oral Pathogens and Cytotoxicity of *Glycyrrhiza uralensis* Extract

**DOI:** 10.3390/plants9070838

**Published:** 2020-07-03

**Authors:** Song-Yi Yang, Yu-Ri Choi, Myung-Jin Lee, Min-Kyung Kang

**Affiliations:** 1 Department and Research Institute of Dental Biomaterials and Bioengineering, Yonsei University College of Dentistry, Seoul 03722, Korea; syyang88@yuhs.ac; 2 Department of Dental Hygiene, Hallym Polytechnic University, Gangwon-do 24210, Korea; cyr@hsc.ac.kr; 3 Department of Dental Hygiene, Division of Health Science, Baekseok University, Chungcheongnam-do 31065, Korea; dh.mjlee@bu.ac.kr; 4 Department of Dental Hygiene, Hanseo University, Chungcheongnam-do 31962, Korea

**Keywords:** antimicrobial activity, cytotoxicity, dental materials, *Glycyrrhiza uralensis*, *Streptococcus mutans*, *Candida albicans*

## Abstract

We aimed to evaluate the antimicrobial effects of *Glycyrrhiza uralensis* extract on *Streptococcus mutans* and *Candida albicans* and its biocompatibility for dental applications. The antimicrobial activity of the *G. uralensis* extracts at concentrations of 50, 100, 150, and 200 µg/mL was assessed using agar disk diffusion tests, counting the total number of colony-forming units (CFUs), spectrophotometric growth inhibitory assays, and microbial morphology observations using scanning electron microscopy (SEM; Merin, Carl Zeiss, Oberkochen, Germany). We measured the polyphenol and flavonoid contents of *G. uralensis* extracts using ultraviolet–visible spectrometry and the cytotoxicity of these extracts using an MTT (3-(4,5-Dimethylthiazol-2-yl)-2,5-diphenyltetrazolium bromide) assay. We identified that *G. uralensis* extracts had significant antimicrobial effects against *S. mutans* and *C. albicans*. The optical density of the experimental groups significantly decreased compared with that of the control group. SEM images revealed that the *G. uralensis* extract affected the morphology and density of *S. mutans* and *C. albicans*. The extract concentration of flavonoids, but not polyphenols, increased with increasing concentrations of the *G. uralensis* extract. Furthermore, cell viabilities were more than 70% for *G. uralensis* extracts with concentrations of 50 and 100 μg/mL. Naturally derived *G. uralensis* is biocompatible and exhibits an excellent antimicrobial effect against oral pathogens such as *S. mutans* and *C. albicans*. Thus, *G. uralensis* extracts can be used for the development of oral products that treat and prevent oral diseases.

## 1. Introduction

The accumulation of dental plaque around teeth and prosthetics may develop into dental caries or periodontitis. Dental caries develop because of the destructive forces of acid produced by oral bacteria, such as *Streptococcus mutans*, while periodontitis involves an infection of the gums that leads to inflammation and bone loss [1,2]. In patients that use dentures, the attachment of microorganisms to the denture surface can cause denture-related stomatitis. Infection involving *Candida albicans* is the leading cause of denture stomatitis, and this microbe can attach to the surface of prosthetic materials [3,4].

The prevention of dental caries and infections by oral pathogens depends on regular and thorough cleaning of the mouth and dentures. Mouthwash or denture cleansing solutions are widely used for oral hygiene and are particularly useful for physically limited patients. Most commercial mouthwash and denture cleaning solutions are prepared with chemical components that can present non-biocompatible reactions because of their direct contact with the oral tissue [5]. Furthermore, there have been some reports that the chemicals used in denture cleaning procedures could damage acrylic resin materials [6,7].

To identify alternatives to problematic chemical additives in oral cleaning aids, researchers have examined natural products as essential components in mouthwash and denture cleaning solutions [8,9]. Many of these products are harmless to humans when used correctly and can inactivate bacteria, yeast, and spores. *Glycyrrhiza uralensis* Fisch, commonly known as Chinese licorice root, is one of the most popular natural products used in traditional medicines, and its chemical constituents and biological activities have been examined in clinical settings [10,11,12]. The compound glycyrrhizol A, present in the ethanolic extract from *G. uralensis*, has high antibacterial activity against *S. mutans*, and flavonoids in *G. uralensis* inhibit the activity of oral pathogens [12,13,14]. Although components of *G. uralensis* have been explored as antimicrobial agents, the use of *G. uralensis* extracts to prevent or treat oral infections, and the biocompatibility of this plant for application in oral hygiene materials, have not yet been examined.

In this study, we assessed the potential effects of naturally derived *G. uralensis* extracts at various concentrations on the common oral pathogens *S. mutans* and *C. albicans*, based on in vitro evaluation methods for application in antimicrobial oral products.

## 2. Materials and Methods

### 2.1. Preparation of G. uralensis Extract

Roots of *Glycyrrhiza uralensis* (purity 99.7%), cultivated in the Sobaek Mountains located in Gyeongsangnam-do of South Korea, were obtained from an official herbal shop (Chiri-san yakchogol, Gyeongsangnam-do, Korea). Five hundred grams of *G. uralensis* were crushed, immersed in 5 L of 70% methanol solution, and extracted at room temperature (25 ± 1 °C) for 48 h. Then, a dry extract was prepared by filtering the solution (Filter paper #2, Whatman, Maidstone, UK), concentrating it by evaporation in a vacuum evaporator (EYELA, Tokyo, Japan), and then freeze-drying (Freeze Dryer, Ilshin Lab, Gyeonggi-do, Korea) at −55 °C for 48 h. We adjusted the concentration of *G. uralensis* extract to 0, 50, 100, 150, and 200 µg/mL with dimethyl sulfoxide (DMSO, Sigma-Aldrich, St. Louis, MO, USA) for use in an antimicrobial activity test solution.

### 2.2. Microbial Preparation

We examined the antimicrobial effect of the different concentrations of *G. uralensis* extract on *S. mutans* (ATCC 25175) and *C. albicans* (ATCC 10231). *Streptococcus mutans* was cultured in brain heart infusion (BHI, Becton Dickinson and Co., MD, USA) and *C. albicans* in yeast mold (YM, Becton Dickinson and Co., Franklin Lakes, NJ, USA). Both species were subsequently incubated at 37 °C for 24 h under aerobic conditions.

### 2.3. Inhibition Zone Test

We spread a 100 µL microbial culture suspension (1 × 10^4^ cells/mL) of *S. mutans* and *C. albicans* onto BHI and YM agar plates, respectively. We placed five filter paper disks, each with a diameter of 10 mm and thickness of 1 mm, onto the surface of one agar plate with either the *S. mutans* or the *C. albicans* culture, and then deposited 20 µL of the *G. uralensis*/DMSO experimental solution on one of the paper disks, for a total of five treatments (0, 50, 100, 150, and 200 µg/mL extracts) per plate. The plates were incubated for 24 h at 37 °C, and the inhibition zones around each sample were measured with vernier calipers (Mitutoyo, Kawasaki, Japan) with an accuracy of ± 0.01 mm.

### 2.4. Evaluation of Colony-Forming Units (CFUs)

We mixed a 1:1 ratio of *G. uralensis*/DMSO (0, 50, 100, 150, or 200 µg/mL) and microbial culture fluid (1 × 10^5^ cells/mL) for both *S. mutans* and *C. albicans*. Then, we spread 100 μL of this mixture onto BHI (*S. mutans*) or YM (*C. albicans*) agar plates. We incubated the plates for 48 h at 37 °C before determining the number of viable CFUs of *S. mutans* and *C. albicans*. 

### 2.5. Evaluation of Optical Density (OD)

We used measures of the OD of microbial cultures treated with *G. uralensis*/DMSO (0, 50, 100, 150, or 200 µg/mL) to estimate microbial viability. Samples of the microbial cultures were diluted so that the OD_600_ value was 0.4–0.6. We mixed the *G. uralensis* extract solution with the microbial culture fluid in a 9:1 ratio and then incubated the culture at 37 °C for 12 h and 24 h. Estimates of the growth inhibitory effects of the *G. uralensis*/DMSO solution were obtained from OD values that were measured with an ELISA reader (Epoch, BioTeck, Winooski, VT, USA) at 600 nm.

### 2.6. Morphological Observations

In addition to changes in microbial growth and culture formation, we also examined evidence of morphological change with scanning electron microscopy (SEM) analyses. We incubated a 1 mL sample of the *S. mutans* and *C. albicans* suspension (1 × 10^5^ cells/mL) on a 24-well plate at 37 °C for 24 h and then fixed the sample with 2% glutaraldehyde–paraformaldehyde in 0.1 M PBS for at least 30 min at room temperature (25 ± 1 °C). The samples were post-fixed with 1% OsO_4_ dissolved in 0.1 M PBS for 2 h, dehydrated in an ascending gradual series of ethanol, treated with isoamyl acetate, and subjected to critical point drying (LEICA EM CPD300; Leica, Wien, Austria). We then coated the samples with Pt (5 nm) using an ion coater (ACE600; Leica, Wien, Austria) and photographed each sample using SEM (FE-SEM; Merin, Carl Zeiss, Oberkochen, Germany) at an operating voltage of 2 kV.

### 2.7. Measurement of Polyphenol and Flavonoid Contents

To confirm the contents of polyphenols and flavonoids, *G. uralensis* extract powder was dissolved in distilled water to make the concentrations 50, 100, 150, and 200 µg/mL. The solutions were then stored at 37 ± 1 °C in a water bath for 24 h prior to measurements. To analyze the polyphenol content, 650 μL of distilled water was added to 50 μL of the extract solution, followed by the addition of 50 μL of Folin–Denis reagent. The reaction was allowed to continue for 3 min, and then 100 μL of 10% Na_2_CO_3_ solution and 150 μL of distilled water were added for a total volume of 1 mL. After incubation for 1 h, we measured the absorbance at 725 nm using ultraviolet–visible (UV–VIS) spectrophotometry (X-ma 1200 Spectrophotometer, Human Corp., Seoul, Korea). The standard curve (20, 40, 60, 80, and 100 μg/mL) was calculated using a standard gallic acid solution (Sigma-Aldrich, St Louis, MO, USA). 

For the analysis of the flavonoid contents, 1 mL diethylene glycol was added to 100 µL of the *G. uralensis* extract solution (50, 100, 150, or 200 μg/mL), followed by the addition of 100 µL of 1 N NaOH. The solution was stored at 37 °C for 1 h, and then the absorbance was measured at 420 nm using a UV–VIS spectrophotometer. A standard curve (20, 40, 60, 80, and 100 μg/mL) was drawn using naringin (Sigma-Aldrich, St Louis, MO, USA) as a standard material to calculate the content of flavonoids. The content measurement of polyphenols and flavonoids was repeated in five separate experiments.

### 2.8. Cytotoxicity Tests

To prepare the experimental solution for the cytotoxicity test, *G. uralensis* extract powder was dissolved in RPMI 1640 (Gibco Laboratories, Grand Island, NY, USA) cell culture medium to obtain 50, 100, 150, and 200 μg/mL solutions, and these solutions were stored at 37 ± 1 °C in a humidified 5% CO_2_/air environment for 24 h following ISO standard 10993-12 (Biological evaluation of medical devices—Part 12: Sample preparation and reference materials) [15]. As a control for the cytotoxicity, we used RPMI 1640 cell culture medium without *G. uralensis* extract. The MTT assay was conducted according to ISO 10993-5 (Biological evaluation of medical devices—Part 5: Tests for cytotoxicity—in vitro methods) [16]. One hundred microliters of the L929 cell solutions (1 × 10^4^ cells/mL) were seeded on 96-well plate, and stored at 37 ± 1 °C in a humidified 5% CO_2_/air environment. After 24 h, the culture medium in the 96-well plate was removed, and the attached cells on the cell culture plates were post-incubated with 100 μL of experimental and control solutions for 24 h. After the experimental and control solutions were removed, the wells were refilled with 50 μL of 1 mg/mL MTT tetrazolium salts (Sigma, St. Louis, MO, USA) in PBS and kept at 37 ± 1 °C in a dark, humidified 5% CO_2_/air environment for 2 h. The MTT solution was then removed, and the wells were refilled with 100 μL of isopropanol (Sigma, St. Louis, MO, USA) and placed on a shaker for 20 min in a dark environment. The absorbance of each sample was measured at 570 nm using UV–VIS spectrophotometry, and the percentage viability was calculated as cell viability (%) = (OD of treated cell / OD of negative control) × 100. 

Additionally, we used an EVOS FL microscope (EVOS FL, Advanced Microscopy Group USA Ltd., Mill Creek, WA, USA) at 20× magnification to examine the micromorphology of the L929 cells in the 96-well plate after treatment with 100 µL of the *G. uralensis* extract solutions (50, 100, 150, or 200 μg/mL) for 24 h.

### 2.9. Statistical Analysis

All experiments except for the SEM observations were independently performed with five repetitive tests, and data were calculated as averages and standard deviations. All test results from the experimental and control groups were analyzed with one-way analysis of variance (ANOVA; IBM SPSS Statistics 25.0, IBM Co., Armonk, NY, USA) to evaluate the interactions with different concentrations of *G. uralensis* extract. To determine significant differences within various concentrations of *G. uralensis* extract, post hoc analyses were performed with Tukey’s multiple comparison test at a significance level of 0.05 (*p* = 0.05).

## 3. Results

### 3.1. Inhibition Zone

**Figure 1:**
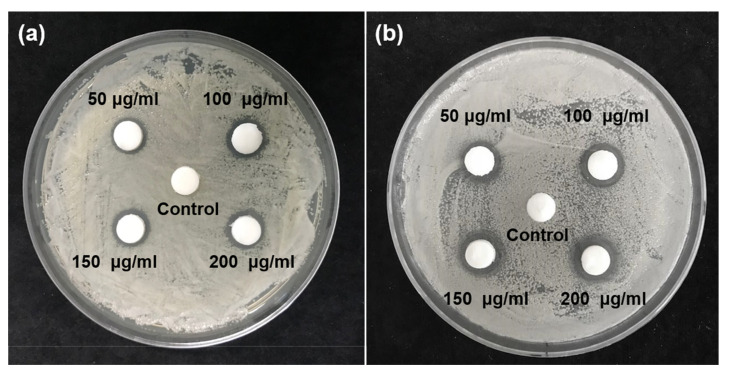
Inhibition zones of different concentrations (50 μg/mL, 100 μg/mL, 150 μg/mL, and 200 μg/mL) of *Glycyrrhiza uralensis* extract on agar plates of (**a**) *Streptococcus mutans* and (**b**) *Candida albicans*.

We found that all concentrations of the *G. uralensis* extract inhibited the growth of *S. mutans* and *C. albicans* colonies (Figure 1). A microbial static ring was observed around the paper disk impregnated with experimental solution. The inhibition zones of *S. mutans* increased in the order: 50 µg/mL (1.00 ± 0.10 mm) < 100 µg/mL (1.07 ± 0.06 mm) < 150 µg/mL (1.13 ± 0.15 mm) < 200 µg/mL (1.17 ± 0.15 mm). Similarly, the inhibition zones of *C. albicans* also increased in following order: 50 µg/mL (1.13 ± 0.15 mm) < 100 µg/mL (1.23 ± 0.06 mm) < 150 µg/mL (1.33 ± 0.15 mm) < 200 µg/mL (1.40 ± 0.17 mm). There were no significant differences among the experimental groups (*p* > 0.05) for both *S. mutans* and *C. albicans*. In contrast, there was no inhibition zone in the control group. 

### 3.2. Colony-Forming Units

All concentrations of the *G. uralensis* extract resulted in a reduction in the number of CFUs for both *S. mutans* and *C. albicans*. The CFUs of *S. mutans* decreased in the order: Control (99.7 ± 13.6) > 50 µg/mL (29.3 ± 9.4) > 100 µg/mL (23.0 ± 6.6) > 150 µg/mL (22.3 ± 5.7) > 200 µg/mL (19.0 ± 8.4). Similarly, the CFUs of *C. albicans* also decreased in the following order: Control (282.3 ± 6.8) > 50 µg/mL (183.5 ± 16.0) > 100 µg/mL (171.5 ± 17.4) > 150 µg/mL (131.3 ± 19.9) > 200 µg/mL (117.5 ± 9.8). The number of CFUs in the experimental groups containing the *G. uralensis* extract was significantly lower compared to the control group (*p* < 0.05).

**Figure 2:**
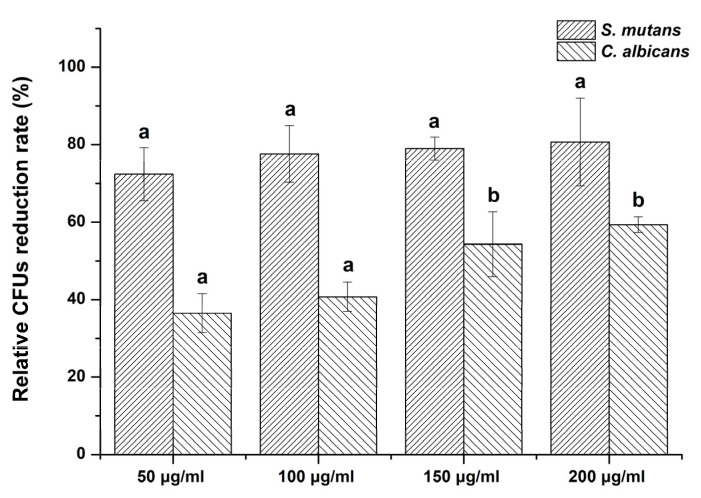
Relative colony-forming unit (CFU) reduction rate of two microbial strains following exposure to *Glycyrrhiza uralensis* extract with concentrations of 50, 100, 150, and 200 µg/mL. The same lowercase letter shows no significant difference in CFU reduction rate between the groups of a given microbe (*p* > 0.05).

When the CFUs of the control were set at 100%, the CFU reduction rate for *S. mutans* did not differ significantly among the experimental groups (*p* > 0.05) (Figure 2). The CFU reduction rate for *C. albicans* increased with increasing concentrations of *G. uralensis* extract. There were no significant differences in the CFU reduction rates between 150 μg/mL vs. 200 μg/mL samples (*p* > 0.05) and between the 50 μg/mL vs. 100 μg/mL samples (*p* > 0.05). In all experimental groups, the CFU reduction rate was higher in *S. mutans* than in *C. albicans*.

### 3.3. Growth Inhibitory Effect

We found that the OD value of the experimental groups containing *G. uralensis* extract significantly decreased with *S. mutans* and *C. albicans* compared with the control group (*p* < 0.05). However, the OD values for both *S. mutans* and *C. albicans* did not differ significantly among the experimental groups at each time point (*p* > 0.05). 

**Figure 3:**
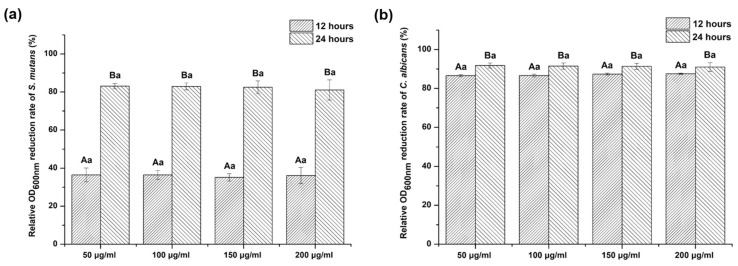
Relative optical density (OD_600nm_) value reduction rate of (**a**) *Streptococcus mutans* and (**b**) *Candida albicans* exposed to *Glycyrrhiza uralensis* extract with concentrations of 50, 100, 150, and 200 µg/mL at two different times. The same lowercase letters show no significant difference in relative OD_600nm_ reduction rate between the experimental groups at each time point (*p* > 0.05). The different uppercase letters indicate significant differences in relative OD_600nm_ reduction rate between 12 and 24 h for each experimental group (*p* < 0.05).

When the OD value of the control was set at 100%, the relative OD reduction rate for both *S. mutans* and *C. albicans* did not differ significantly among the experimental groups at each cultivation time (*p* > 0.05) (Figure 3). The cultivation time significantly affected the OD reduction rate for both *S. mutans* and *C. albicans* (*p* < 0.05). The relative OD reduction rate of *C. albicans* was more than 80% within 12 h of cultivation time with the experimental groups. However, *S. mutans* did not show an OD reduction rate as high as *C. albicans* even after 12 h of incubation with the experimental groups.

### 3.4. Characterization of Microbial Morphology

**Figure 4:**
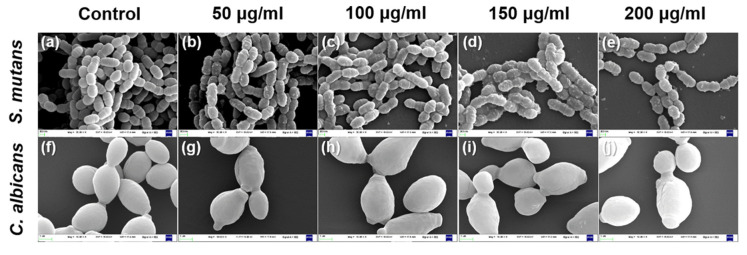
Field-emission scanning electron microscopy images of *Streptococcus mutans* and *Candida albicans* following (**a**,**f**) control and exposure to *Glycyrrhiza uralensis* extract with concentrations of (**b**,**g**) 50, (**c**,**h**) 100, (**d**,**i**) 150, and (**e**,**j**) 200 µg/mL. Upper 20,000×, lower 10,000×.

The microbial morphologies of both control groups showed intact and clear surfaces without debris or lysis (Figure 4). The control group for *S. mutans* showed relatively aggregated chains and differed from the 200 µg/mL group in this respect. Additionally, *S. mutans* cells treated with *G. uralensis* extract showed small debris on the cells and unclear wall bands. The control group for *C. albicans* showed a relatively larger number of yeast cells than the experimental groups containing *G. uralensis* extract. *Candida albicans* cells treated with *G. uralensis* extract showed uneven surfaces and swelling compared with the control group. These results show that *G. uralensis* extract significantly affected the microbial morphology and formation of *S. mutans* and *C. albicans* colonies.

### 3.5. Polyphenol and Flavonoid Contents

The different concentrations of the *G. uralensis* extract were confirmed to contain different concentrations of flavonoids, but similar concentrations of polyphenols (Table 1). We did not observe any significant differences in polyphenols among the different experimental groups (*p* > 0.05). The experimental group with the highest concentration (200 µg/mL) had the highest flavonoid content (*p* < 0.05). However, polyphenol and flavonoid contents in other experimental groups (50, 100, and 150 µg/mL) were not significantly different in each component (*p* > 0.05).

### 3.6. Cytotoxicity

**Figure 5:**
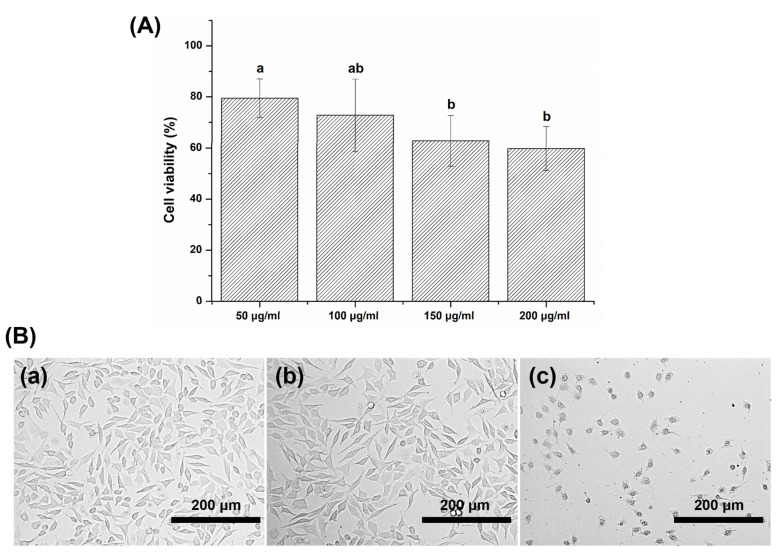
(**A**) Percentage viability of L929 cells exposed to different concentrations of the *Glycyrrhiza* *uralensis* extract in the MTT assay. The same lowercase letters show no significant difference in cell viability (%) between the experimental groups (*p* > 0.05). (**B**(**a**)) L929 cells exposed to negative control, (**B**(**b**)) L929 cells exposed to *G. uralensis* extract at a concentration of 50 µg/mL, and (**B**(**c**)) L929 cells exposed to *G. uralensis* extract at a concentration of 200 µg/mL for 24 h.

We found that the *G. uralensis* extract, in general, decreased cell viability (Figure 5A). The MTT assay showed that the viability of the L929 cells decreased as the concentration of *G. uralensis* extract increased in the following concentration (µg/mL) order: 50 > 100 > 150 > 200. The experimental solution with 50 µg/mL of *G. uralensis* extract yielded a survival rate of more than 70%, which was higher than that of the 150 and 200 µg/mL groups (*p* < 0.05). However, no significant difference was observed between the results for the experimental solutions with 50 and 100 µg/mL of the extract (*p* > 0.05). 

We also observed that the L929 cells in the 50 µg/mL group showed a morphological appearance (typical stellate) similar to that of the control group (Figure 5B). However, in treatments with higher concentrations of *G. uralensis* extract, the L929 cells showed an abnormal response. The cells in the 200 μg/mL group were rounded and lacked the original structure that was observed in the control group.

## 4. Discussion

The results of this study demonstrate the promise of *G. uralensis* extract for the treatment and prevention of oral diseases caused by the common oral pathogens *S. mutans* and *C. albicans*. These pathogens have long been recognized as the primary pathogens responsible for dental caries and candidal stomatitis. Despite attempts to use antimicrobial agents to treat and prevent oral diseases caused by these pathogens, decay and infection in the oral cavity remain a problem for humans [17,18,19,20]. *Glycyrrhiza uralensis* (Leguminosae) has been considered to have anti-inflammatory, anti-allergic, and anti-viral properties that can be attributed to its major bioactive components, such as glycyrrhizin of the saponin family and flavonoid compounds [21,22,23,24,25]. Previous studies have also demonstrated that *G. uralensis* exhibits considerable antibacterial activity [5,12,26]. However, most of these studies have only tested for antibacterial properties, and no studies have investigated their potential or safety as raw materials for improving oral health [27]. Here, we demonstrated the antimicrobial effect of various concentrations of *G. uralensis* extract on oral pathogens and the biocompatibility of this product for use in dental materials.

To confirm the antimicrobial efficacy of *G. uralensis* extract against *S. mutans* and *C. albicans*, the characteristics of microbial growth on the agar plate were observed. No inhibition zone was observed for the control group. Meanwhile, experimental groups containing four different concentrations of the *G. uralensis* extract showed a clear inhibition zone around the test specimens impregnated with the experimental solution. This was compared with the results of the inhibition zone on the *Staphylococcus aureus* agar plate of *Rubus coreanus* extract [26,28]. The *G. uralensis* extract applied in this study had superior antimicrobial properties, even though the concentration of the *G. uralensis* extract was lower than that of *R. coreanus* extract. In our study, even low concentrations of *G. uralensis* extract effectively inhibited the growth of *S. mutans* and *C. albicans* on agar plates.

The disk diffusion method has a limited ability to quantify the degree of antimicrobial activity [29]. Therefore, we confirmed the antimicrobial effect *G. uralensis* extract by counting the number of CFUs. We found that *G. uralensis* extract had better antibacterial activity against *S. mutans* than against *C. albicans*, but there was no significant difference between the CFU values for the different concentrations of the experimental group.

OD measurement by culturing in an appropriate medium was used for the quantification of the microbial growth pattern in the *G. uralensis* extract [30]. Hence, it was used in this study to determine the influence of *G. uralensis* extract on the control of the proliferation of microbes in each culture medium. As shown in Figure 3, *S. mutans* and *C. albicans* cultures treated with various concentrations of *G. uralensis* extract showed a significantly lower OD than those treated with the control. However, there were no significant differences in OD values between concentrations of the extract. These results were similar to the measures of growth inhibition from the agar plate experiments.

We examined the effects of the *G. uralensis* extract on the morphology of *S. mutans* and *C. albicans* using SEM. The control group for *S. mutans* showed the bacterial form to be distinct and clustered in long chains, along with a high density, while the experimental groups showed the formation of short chains and a low density of aggregates. In addition, the control group for *C. albicans* showed a high density of spherical aggregates, while aggregates in the experimental group showed a relatively low density and uneven surface. These results were similar to the effect of *xanthorrhizol*, an antibacterial substance derived from the rhizome of an indigenous Indonesian medicinal plant, on the morphology of *S. mutans* [31]. The observed antimicrobial effect of *G. uralensis* extract may be driven by the extract’s negative effect on the morphology of the oral pathogens, thus hindering their normal growth.

We examined the presence of possible antimicrobial components present in *G. uralensis* extract, including phenols and flavonoids. Phenolic compounds are secondary metabolites present in plant systems. These compounds are responsible for physiological activation functions such as antioxidant and antibacterial activities [32,33]. We found no significant differences in detected phenol content in distilled water among different concentrations of *G. uralensis* extract. However, we did find that the released flavonoid content in distilled water was significantly different among concentrations of *G. uralensis* extract. Previous research has confirmed that phenolic compounds possess antibacterial properties and reported that the microbial growth was in proportion to the content of phenolic compounds contained in plant extracts [34,35]. The polyphenols and flavonoids contained in *G. uralensis* extract may have resulted in the antimicrobial activity of this compound that was observed in this study.

Although many plant compounds may possess antimicrobial activities, those compounds must be safe for human consumption and frequent exposure within the oral cavity [36,37]. In this study, the effect of various concentrations of *G. uralensis* extract on cell viability was confirmed using in vitro methods. The MTT assay is considered when selecting tests for the biological evaluation of medical devices [38]. The biological risk from *G. uralensis* extract can be estimated by the cytotoxicity tests. L929 cells are used for the screening or ranking of the cytotoxicity potential of experimental materials. In addition, they are known to have similar responses to human gingival fibroblasts [39]. According to the international standard for biocompatibility evaluation methods, L929 cells were treated with four concentrations of *G. uralensis* extract, and then cell viability was evaluated using MTT quantitative analysis. We found that *G. uralensis* extract caused very weak cytotoxicity for L929 cells at concentrations between 150 and 200 μg/mL, whereas exposure to *G. uralensis* extract concentrations of 50 and 100 μg/mL resulted in cell viabilities of 70% or more. This level of cytotoxicity at the lower concentrations meets the international guidelines for use in products intended for human consumption. In addition, we did not observe any significant changes in cell morphology after exposure to *G. uralensis* extract concentrations of 50 and 100 μg/mL. 

According to our results, we concluded that *G. uralensis* extract with concentrations of up to 100 μg/mL is biocompatible according to ISO 10993-5 [16]. Furthermore, concentrations of *G. uralensis* extract over 100 μg/mL did not have significantly higher antimicrobial effects. Therefore, we hypothesize that *G. uralensis* extracts at concentrations of 100 μg/mL exhibit an antimicrobial effect that does not differ significantly from that exhibited by > 100 μg/mL of *G. uralensis* extract, without showing a negative effect on biocompatibility. It is presumed to be suitable for patients who want to treat and prevent oral diseases associated with dental caries and denture stomatitis. Future studies should examine the practical use of this material by conducting studies on the long-term antimicrobial effects and biocompatibility of *G. uralensis* extract. 

The results presented here are limited to the verification of the antimicrobial activity of *G. uralensis* extract on oral disease-causing bacteria or fungi and the possible biocompatibility of this natural product. When using the *G. uralensis* extract as a mouthwash, the period observed in this study can be considered to be longer than the recommended time by the manufacturer. Nevertheless, the reason why the observation time was longer than the actual clinical application time is that antimicrobial action may occur for a relatively long time if the *G. uralensis* extract remains in the oral cavity after using the mouthwash. This study was conducted by selecting two types of microorganisms that cause frequent oral diseases, *S. mutans* and *C. albicans,* which were also used in previous studies to confirm the antimicrobial effects of newly developed dental materials [17,18,19,20]. To overcome these limitations, further studies are needed to consider shorter application periods and various microbial species for scientifically identified *G. uralensis* extract with standard antimicrobial agents. In addition, to verify the utility of this product when incorporated into oral products, studies that assess whether the antimicrobial potency is maintained for a long period and if the antimicrobial properties of *G. uralensis* extracts are diminished when incorporated with ingredients used in mouthwashes or denture cleansers are needed. Nevertheless, some of these oral products could be produced using naturally derived extracts as alternative raw materials in the future because it is biocompatible and exhibits an excellent antimicrobial effect against oral pathogens such as *S. mutans* and *C. albicans*.

## 5. Conclusions

The *G. uralensis* extract showed significant antimicrobial effects compared with the control group, with no significant difference among four different concentrations of *G. uralensis* extract against two oral pathogens, *S. mutans* and *C. albicans*. In addition, *G. uralensis* extract with concentrations of 50 and 100 μg/mL showed a cell viability of 70% or more, which meets the international standard. The *G. uralensis* extract at a concentration of 100 μg/mL was considered a biocompatible, naturally derived extract and also exhibited excellent antimicrobial effects against *S. mutans* and *C. albicans*. 

## Figures and Tables

**Table 1:** Polyphenol and flavonoid content of *Glycyrrhiza uralensis* extract.

Experimental Group	Polyphenol Content (μg/mL)	Flavonoid Content (μg/mL)
50 μg/mL	13.1 ± 3.4 ^a^	21.7 ± 1.6 ^a^
100 μg/mL	13.8 ± 1.4 ^a^	21.8 ± 1.0 ^a^
150 μg/mL	16.2 ± 0.8 ^a^	21.8 ± 0.9 ^a^
200 μg/mL	17.7 ± 3.9 ^a^	25.0 ± 1.6 ^b^

Different superscript letters denote significant differences between the experimental groups in each component (*p* < 0.05).

## References

[1] Hammad M., Sallal A.K., Darmani H. (2007). Inhibition of Streptococcus mutans adhesion to buccal epithelial cells by an aqueous extract of Thymus vulgaris. Int. J. Dent. Hyg..

[2] Rosan B., Lamont R.J. (2000). Dental plaque formation. Microbes Infect..

[3] Cateau E., Berjeaud J.M., Rodier M.H., Imbert C. (2008). Fungal biofilm inhibition by a component naturally produced by Candida albicans yeasts growing as a biofilm. Int. J. Antimicrob. Agents.

[4] Kojic E.M., Darouiche R.O. (2004). Candida infections of medical devices. Clin. Microbiol. Rev..

[5] Kim R.W., Lee S.Y., Kim S.G., Heo Y.R., Son M.K. (2016). Antimicrobial, Antioxidant and Cytotoxic Activities of Dendropanax morbifera Léveille extract for mouthwash and denture cleaning solution. J. Adv. Prosthodont..

[6] Jagger D.C., Harrison A. (1995). Denture cleansing—The best approach. Br. Dent. J..

[7] Lee H., Li C., Chang H., Yang Y., Wu J. (2011). Effects of different denture cleaning methods to remove Candida albicans from acrylic resin denture based material. J. Dent. Sci..

[8] Almas K., Skaug N., Ahmad I. (2005). An in vitro antimicrobial comparison of miswak extract with commercially available non-alcohol mouthrinses. Int. J. Dent. Hyg..

[9] Kim S.J., Kim S.J., Hong M., Choi H.G., Kim J.A., Lee S. (2016). Investigation of selective inhibitory effects of glycyrol on human CYP 1A1 and 2C9. Xenobiotica.

[10] Hatano T., Aga Y., Shintani Y., Ito H., Okuda T., Yoshida T. (2000). Minor flavonoids from licorice. Phytochemistry.

[11] Tsukiyama R., Katsura H., Tokuriki N., Kobayashi M. (2002). Antibacterial activity of licochalcone a against spore-forming bacteria. Antimicrob. Agents Chemother..

[12] He J., Chen L., Heber D., Shi W., Lu Q.Y. (2006). Antibacterial compounds from Glycyrrhiza uralensis. J. Nat. Prod..

[13] Chen Y., Agnello M., Dinis M., Chien K.C., Wang J., Hu W., Shi W., He X., Zou J. (2019). Lollipop containing Glycyrrhiza uralensis extract reduces Streptococcus mutans colonization and maintains oral microbial diversity in Chinese preschool children. PLoS ONE.

[14] Villinski J.R., Bergeron C., Cannistra J.C., Gloer J.B., Coleman C.M., Ferreira D., Azelmat J., Grenier D., Gafner S. (2014). Pyrano-isoflavans from Glycyrrhiza uralensis with antibacterial activity against Streptococcus mutans and Porphyromonas gingivalis. J. Nat. Prod..

[15] ISO (2012). ISO 10993-12: 2012 Biological Evaluation of Medical Devices—Part 12: Sample Preparation and Reference Materials.

[16] ISO (2009). ISO 10993-5: 2009 Biological Evaluation of Medical Devices—Part 5: Tests for In Vitro Cytotoxicity.

[17] Campos K.P.L., Viana G.M., Cabral L.M., Portela M.B., Hirata Junior R., Cavalcante L.M., Lourenço E.J.V., Telles D.M. (2020). Self-cured resin modified by quaternary ammonium methacrylates and chlorhexidine: Cytotoxicity, antimicrobial, physical, and mechanical properties. Dent. Mater..

[18] Guandalini Cunha B., Duque C., Sampaio Caiaffa K., Massunari L., Araguê Catanoze I., Dos Santos D.M., de Oliveira S.H.P., Guiotti A.M. (2020). Cytotoxicity and antimicrobial effects of citronella oil (Cymbopogon nardus) and commercial mouthwashes on S. aureus and C. albicans biofilms in prosthetic materials. Arch. Oral Biol..

[19] Falsetta M.L., Klein M.I., Colonne P.M., Scott-Anne K., Gregoire S., Pai C.H., Gonzalez-Begne M., Watson G., Krysan D.J., Bowen W.H. (2014). Symbiotic relationship between Streptococcus mutans and Candida albicans synergizes virulence of plaque biofilms in vivo. Infect. Immun..

[20] Lu Z., Rong K., Li J., Yang H., Chen R. (2013). Size-dependent antibacterial activities of silver nanoparticles against oral anaerobic pathogenic bacteria. J. Mater. Sci. Mater. Med..

[21] Afreen F., Zobayed S.M., Kozai T. (2006). Melatonin in Glycyrrhiza uralensis: Response of plant roots to spectral quality of light and UV-B radiation. J. Pineal Res..

[22] Ahn E.Y., Shin D.H., Baek N.I., Oh J.A. (1998). Isolation and identification of antimicrobial activity substance from Glycyrrhiza uralensis FISCH. Korean J. Food Sci. Technol..

[23] Asl M.N., Hosseinzadeh H. (2008). Review of pharmacological effects of *Glycyrrhiza* sp. and its bioactive compounds. Phytother. Res..

[24] Tsuge A., Hisaka S., Hayashi H., Nose M. (2020). Effect of hot water extract of a glycyrrhizin-deficient strain of Glycyrrhiza uralensis on contact hypersensitivity in mice. J. Nat. Med..

[25] Zhou Y., Jiao Y., Sun Y., Gao S. (2020). In vitro production and distribution of flavonoids in Glycyrrhiza uralensis Fisch. J. Food Sci. Technol..

[26] Lee J.W., Ji Y.J., Yu M.H., Bo M.H., Seo H.J., Lee S.P., Lee I.S. (2009). Antimicrobial effect and resistant regulation of Glycyrrhiza uralensis on methicillin-resistant Staphylococcus aureus. Nat. Prod. Res..

[27] Gafner S., Bergeron C., Villinski J.R., Godejohann M., Kessler P., Cardellina J.H., Ferreira D., Feghali K., Grenier D. (2011). Isoflavonoids and coumarins from Glycyrrhiza uralensis: Antibacterial activity against oral pathogens and conversion of isoflavans into isoflavan-quinones during purification. J. Nat. Prod..

[28] Park C.G., Bang K.H., Lee S.E., Cha M.S., Seong J.S., Park S.U., Seong N.S. (2001). Antimicrobial effect of various medicinal herb on Staphylococcus aureus. Korean J. Med. Crop Sci..

[29] Balouiri M., Sadiki M., Ibnsouda S.K. (2016). Methods for in vitro evaluating antimicrobial activity: A review. J. Pharm. Anal..

[30] Beyth N., Domb A.J., Weiss E.I. (2007). An in vitro quantitative antibacterial analysis of amalgam and composite resins. J. Dent..

[31] Rukayadi Y., Kwan Hwang J.K. (2007). The effects of xanthorrhizol on the morphology of Candida cells examined by scanning electron microscopy. Microbiol. Indones..

[32] Ji S., Li Z., Song W., Wang Y., Liang W., Li K., Tang S., Wang K., Qiao X., Zhou D. (2016). Bioactive Constituents of Glycyrrhiza uralensis (Licorice): Discovery of the Effective Components of a Traditional Herbal Medicine. J. Nat. Prod..

[33] Tao W.W., Duan J.A., Yang N.Y., Tang Y.P., Liu M.Z., Qian Y.F. (2012). Antithrombotic phenolic compounds from Glycyrrhiza uralensis. Fitoterapia.

[34] Mitani T., Ota K., Inaba N., Kishida K., Koyama H.A. (2018). Antimicrobial Activity of the Phenolic Compounds of Prunus mume against Enterobacteria. Biol. Pharm. Bull..

[35] Nitiema L.W., Savadogo A., Simpore J., Dianou D., Traore A.S. (2012). In vitro antimicrobial activity of some phenolic compounds (coumarin and quercetin) against gastroenteritis bacterial strains. Int. J. Microbiol. Res..

[36] Padovani G.C., Feitosa V.P., Sauro S., Tay F.R., Durán G., Paula A.J., Durán N. (2015). Advances in Dental Materials through Nanotechnology: Facts, Perspectives and Toxicological Aspects. Trends Biotechnol..

[37] Shahi S., Özcan M., Maleki Dizaj S., Sharifi S., Al-Haj Husain N., Eftekhari A., Ahmadian E. (2019). A review on potential toxicity of dental material and screening their biocompatibility. Toxicol. Mech. Methods.

[38] Geurtsen W., Lehmann F., Spahl W., Leyhausen G. (1998). Cytotoxicity of 35 dental resin composite monomers/additives in permanent 3T3 and three human primary fibroblast cultures. J. Biomed. Mater. Res..

[39] Jevremović D., Kojić V., Bogdanović G., Puškar T., Eggbeer D., Thomas D., Williams R. (2011). A selective laser melted Co-Cr alloy used for the rapid manufacture of removable partial denture frameworks: Initial screening of biocompatibility. J. Serb. Chem. Soc..

